# Identification and Repurposing of Trisubstituted Harmine Derivatives as Novel Inhibitors of *Mycobacterium tuberculosis* Phosphoserine Phosphatase

**DOI:** 10.3390/molecules25020415

**Published:** 2020-01-19

**Authors:** Elise Pierson, Marie Haufroid, Tannu Priya Gosain, Pankaj Chopra, Ramandeep Singh, Johan Wouters

**Affiliations:** 1 Laboratoire de Chimie Biologique Structurale (CBS), Namur Medicine and Drug Innovation Center (NAMEDIC), Namur Research Institute for Life Sciences (NARILIS), University of Namur (UNamur), B-5000 Namur, Belgium; 2 Tuberculosis Research Laboratory, Translational Health Science and Technology Institute, Faridabad 121001, Haryana, India

**Keywords:** *M. tuberculosis*, phosphoserine phosphatase, SerB2, 2,7,9-trisubstituted harmine derivatives

## Abstract

*Mycobacterium tuberculosis* is still the deadliest bacterial pathogen worldwide and the increasing number of multidrug-resistant tuberculosis cases further complicates this global health issue. *M. tuberculosis* phosphoserine phosphatase SerB2 is a promising target for drug design. Besides being a key essential metabolic enzyme of the pathogen’s serine pathway, it appears to be involved in immune evasion mechanisms. In this work, a malachite green-based phosphatase assay has been used to screen 122 compounds from an internal chemolibrary. Trisubstituted harmine derivatives were found among the best hits that inhibited SerB2 activity. Synthesis of an original compound helped to discuss a brief structure activity relationship evaluation. Kinetics experiments showed that the most potent derivatives inhibit the phosphatase in a parabolic competitive fashion with apparent inhibition constants ($K_{i}$) values in the micromolar range. Their interaction modes with the enzyme were investigated through induced fit docking experiments, leading to results consistent with the experimental data. Cellular assays showed that the selected compounds also inhibited *M. tuberculosis* growth in vitro. Those promising results may provide a basis for the development of new antimycobacterial agents targeting SerB2.

## 1. Introduction

Tuberculosis (TB), a disease caused by *Mycobacterium tuberculosis* (Mtb), is still a matter of concern for the World Health Organization (WHO). Each year, an estimated 10 million people fall ill with TB. Although this number has been stable in recent years and the number of casualties is slowly decreasing, drug-resistant cases are on the rise [1,2,3]. To achieve its final goal, a TB-free world by 2035, the WHO invites researchers to find new diagnostics tools, drug-targets, scaffolds and vaccines [4].

To this end, many enzymes from Mtb metabolic pathways, such as secretory tyrosine phosphatases (MptpA, MptpB) or the inosine 5${}^{\prime }$-monophosphate dehydrogenase (GuaB2), have been identified and characterized [5,6,7,8]. Many amino acid biosynthetic pathways, including those of arginine and methionine, have also been proposed as promising therapeutic targets given their essential role in bacterial growth [9,10,11]. One recent focus is on the serine biosynthetic pathway which leads to the production of the non-essential amino acid L-serine [12]. The latter is the source for the production of proteins, the one-carbon metabolism, other amino acids like glycine and tryptophan and phospholipids turnover [13,14,15,16]. Three different enzymes catalyze the three steps involved in the transformation of 3-phosphoglycerate into L-serine. The first enzyme is a phosphoglycerate dehydrogenase (SerA, EC 1.1.1.95) which oxidizes 3-phosphoglycerate into 3-phosphohydroxypyruvate [17]. The latter is transformed in *O*-phospho-L-serine by the phosphoserine aminotransferase (SerC, EC 2.6.1.52) [18]. Finally, a phosphoserine phosphatase (SerB2, EC 3.1.3.3) catalyzes the last irreversible step, the dephosphorylation of *O*-phospho-L-serine into L-serine [19].

Shree et al. [20] showed that Mtb phosphoserine phosphatase SerB2 is secreted within the cytosol of infected macrophages and initiates a dramatic cascade of dephosphorylation. Those events lead to the down-regulation of the immune mediator interleukin 8, which could result in the lack of immune response from the infected host. On the other hand, dephosphorylation of cofilin induces cytoskeletal rearrangements in the macrophage, helping invasion and intracellular survival of the pathogen. SerB2 has further been validated as a drug target and a few inhibitors are already described in the literature [20,21,22].

In this context, this work aims to repurpose molecules from our in-house chemical library and evaluate them for their ability to inhibit SerB2’s activity [23]. The strategy to repurpose already existing drugs and assess their efficiency on Mtb can save time in the drug discovery process and has already led to successful outcomes [24,25,26,27]. Clofazimine, part of the regimen therapy against leprosy is now in the treatment regimen of multidrug-resistant TB and was shown to be active against SerB2 [20,28,29,30]. Anti-inflammatory drugs, like indomethacin or other non-steroidal anti-inflammatory drugs, can also be co-administrated with anti-TB chemotherapy to induce a synergistic effect on the infection site [31,32,33,34].

Our chemical library is enriched with a great range of chemical scaffolds synthesized as part of drug discovery and medicinal chemistry programs from our group [23]. A basic set of a hundred molecules was selected and screened on purified SerB2. Compounds inhibiting the phosphatase activity of the enzyme were considered hits. Interestingly, what emerged out of the screening process was a set of molecules derived from harmine, a natural β-carboline alkaloid obtained from *Peganum harmala*. Such compounds are already known to have many biological activities such as antitumor, antifungal and antibacterial properties among others [35,36,37]. Their specific activity against mycobacterial strains and especially Mtb was assessed in a few papers [38,39,40]. Herein we report the inhibition of SerB2 by trisubstituted harmine derivatives that were originally developed during an anticancer drug discovery program [41,42,43,44,45,46]. Novel activity for those derivatives will be shown in this article.

## 2. Results and Discussion

### 2.1. Screening and Identification of Inhibitors

**Figure 1:**
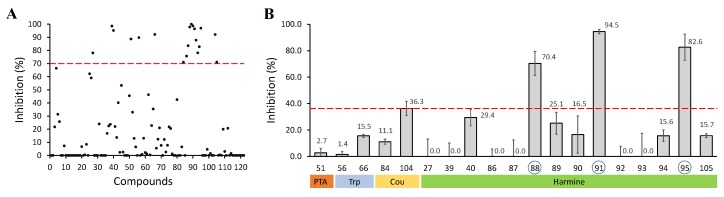
(**A**) Evaluation of 122 compounds for their ability to inhibit SerB2’s phosphatase activity at 100 μM in presence of 0.2 mM *O*-phospho-L-serine. The data depicted are averaged from two independent experiments. (**B**) Evaluation of the 19 primary hits for their ability to inhibit SerB2’s phosphatase activity at 10 μM in presence of 0.2 mM *O*-phospho-L-serine. The data depicted are averaged from three independent experiments ± S.E. PTA: phenyl thiazolamine. Trp: tryptophane. Cou: coumarin.

**Figure 2:**
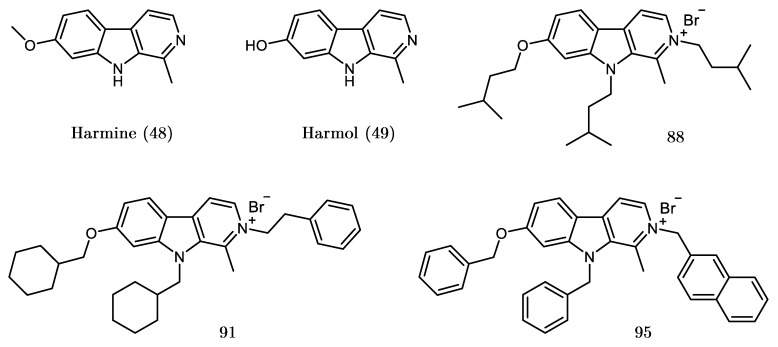
Structures of harmine, harmol and the three 2,7,9-trisubstituted harmine derivatives highlighted in this study.

SerB2 is known to catalyze the hydrolysis *O*-phospho-L-serine into L-serine. During this reaction, the inorganic phosphate (Pi) that is released into the medium can be photometrically determined using a malachite green-based reagent to evaluate the activity of the enzyme [47,48]. Subsequent enzymatic assays were performed in the presence of 122 different therapeutically intended compounds made available by the NAMEDIC research center to identify new anti-TB agents targeting SerB2 (Figure 1A). The preliminary screening was performed at 100 μM in a pH 7.4 reaction media containing 200 μM *O*-phospho-L-serine, 5 mM Mg${}^{2+}$ and 5% dimethyl sulfoxide (DMSO). In total, 19 compounds inhibited the phosphatase activity of SerB2 by at least 70% and were considered to be primary hits. Among them were a phenyl thiazolamine derivative, two tryptophane derivatives, two coumarin derivatives and 14 harmine derivatives. The primary hits were further tested against SerB2 at 10 μM in three independent reactions. As depicted in Figure 1B, compounds **88**, **91** and **95** (Figure 2) are of particular interest since they showed inhibition percentages at least twice as high as the rest of the hits.

A closer look at the structures of the 14 primary harmine-derived hits (Table 1) revealed that they share a common 7,9- or 2,7,9-substituted 7-oxy-1-methyl-β-carboline nucleus. The substitution of this scaffold appears to be essential for the inhibition of SerB2, as emphasized by the inactivity of harmine and harmol (**48** and **49**, Figure 2) in the screening at 100 μM. Table 1 shows the respective R${}^{7/9/2}$ substituents and the inhibition percentages at 10 μM of compounds **88**, **91** and **95** and other structurally related hits. With the exception of compound **93**, all the primary hits are 2,7,9-trisubstituted, which suggests an important role for the positive charge carried by the N${}^{2}$ atom. It can also be noted that the substituents are generally bulky aliphatic or aromatic groups. In order to rationalize their effect, we chose to describe them by their Hansch hydrophobic parameter π [49], calculated using Molinspiration online tool. Knowing that highly hydrophobic substituents possess a large π value, it can be observed that the inhibition efficiency increases with the hydrophobicity of the R${}^{2}$ substituent in each of the three A, B and C sets (Table 1). The same trend also applies to the size of the substituents. On the contrary, derivatives with small polar R${}^{2}$ groups (**86**, **87**, **89**) are mostly inactive. Compound **89** however still shows moderate activity but it could be due to the R${}^{7}$ and R${}^{9}$ methyl-cyclohexyl substituents. The latter seem to improve the inhibition efficiency when comparing compounds of the C set (**89**, **90**, **91**) with their respective counterparts of the A set (**86**, **92**, **94**). Once again, the effect could be related to the higher hydrophobicity of the cyclohexyl ring but also its greater flexibility.

### 2.2. Design, Synthesis and Evaluation of a New Derivative

Compounds **95** and **91** showed the highest inhibition percentages of the test and both of them possess an aromatic R${}^{2}$ substituent. While the difference between the effect of aromatic and aliphatic R${}^{7}$/R${}^{9}$ substituents was highlighted in the previous paragraph, we wanted to verify whether the aromatic feature on position 2 was required to achieve a high inhibition of SerB2. In this view, we designed compound **124**, choosing a N${}^{2}$-ethylcyclohexyl group (π=1.12) to replace the N${}^{2}$-ethylbenzyl group of compound **91**. The two moieties have a relatively similar size, with the ethylcyclohexyl lacking the ability to form aromatic-aromatic interactions and being more flexible.

**Scheme 1:**
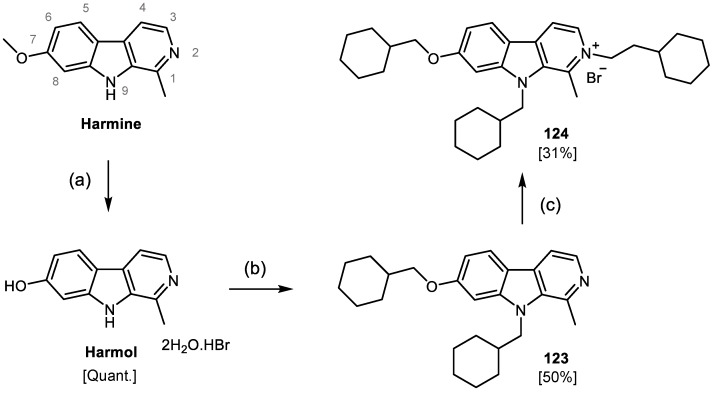
Synthesis of target molecule **124**. (a) HBr, acetic acid, reflux, 72 h; (b) Bromomethyl cyclohexane (2 eq), KOH (5 eq), *N,N*-dimethylformamide (DMF), room temperature, 48 h; (c) 1-bromo-2-cyclohexylethane (10 eq), tetrahydrofuran (THF), microwave, 155 °C, 6 h.

Compound **124** was synthesized according to a published procedure for the synthesis of mono-, di- and trisubstituted harmine derivatives [42,43]. The strategy is depicted in Scheme 1 and starts with the demethylation of harmine to harmol in acidic conditions at reflux. Compound **123** was obtained by a simultaneous N${}^{9}$/O${}^{7}$- alkylation of harmol in presence of 1-bromomethyl cyclohexane and potassium hydroxide in *N,N*-dimethylformamide (DMF). A final N${}^{2}$-alkylation of compound **123** in tetrahydrofuran (THF) using 10 equivalents of 1-bromo-2-cyclohexylethane yielded compound **124**.

After purification by flash chromatography and characterization, the effect of compound **124** was tested at 10 μM on SerB2 using the aforementioned assay. From three independent reactions, we obtained an averaged inhibition percentage of 93.5 ± 1.8%. As the result for compound **91** was 94.5 ± 1.4%, it can be concluded that the aromaticity of the R${}^{2}$ group does not influence the extent of inhibition and that its direct environment upon binding is probably not made of aromatic residues.

### 2.3. Kinetics Assay and Determination of the Inhibition Mode

**Figure 3:**
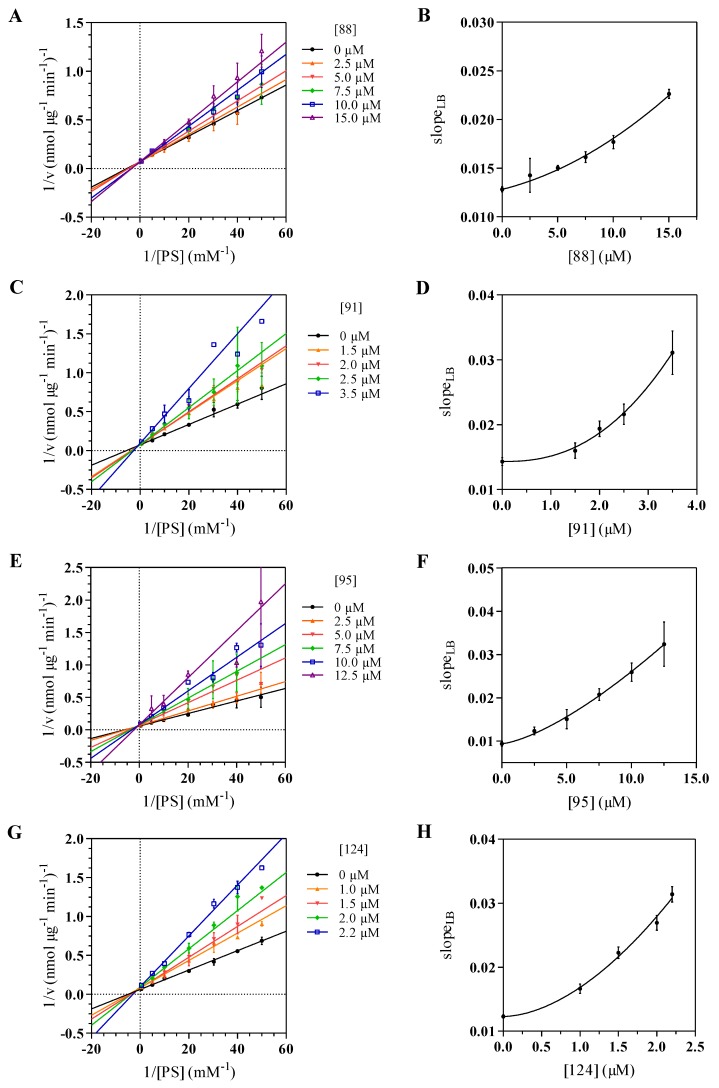
(**A**,**C**,**E**,**G**) Double reciprocal plots of SerB2 inhibition by compounds **88**, **91**, **95** and **124** at various fixed micromolar concentrations in presence of 0 to 2 mM *O*-phospho-L-serine (PS). The data depicted are averaged from two independent experiments ± S.E.; (**B**,**D**,**F**,**H**) Slope replots of double-reciprocal plots A, C, E and G versus inhibitor concentration ± S.E. on slope calculations. Solid line: fitted Hill-type function (Equation (3)).

The inhibition modes of SerB2 by the three best harmine derivatives hits (**88**, **91**, **95**) and the newly synthesized derivative **124** were investigated through enzyme kinetics experiments. Initial velocities were measured with *O*-phospho-L-serine concentrations ranging from 0 to 2 mM at fixed inhibitor concentrations. The resulting double-reciprocal Lineweaver-Burk plots are shown in Figure 3A,C,E,G and were used to judge the binding mechanism of the selected compounds to SerB2. For all four compounds, the double-reciprocal plots yielded straight lines intersecting on the ordinate. These patterns suggest a competitive inhibition mechanism, with the apparent Michaelis constant (K${}_{m}$) increasing with inhibitor concentration and the maximum velocity (V${}_{max}$) remaining unchanged. As inhibitors can be further classified as linear, hyperbolic or parabolic, slopes of the double-reciprocal plots (slope${}_{LB}$) were plotted versus inhibitor concentration to gain further insight into the inhibition mechanism (Figure 3B,D,F,H). The curved-up profiles obtained for all four compounds reveal a parabolic inhibition of SerB2. Such behavior suggests a complex non-linear competitive inhibition type where more than one molecule of inhibitor can bind to the enzyme [50,51].

In an effort to quantify the inhibitory effect, the inhibition constants ($K_{i}$) of the four derivatives were evaluated by fitting Equation (3) (see Section 4) to the data of the slope replots. This equation is based on the Hill-type inhibition model introduced by Cao et al. [52] and used to describe parabolic inhibition [53,54]. While the $K_{i}$ parameters given by the model are rather apparent values since they include the intrinsic constants for the binding of each molecule, they are useful for comparing the relative potencies of the inhibitors.

Good fits were obtained for each compound and $K_{i}$ values in the micromolar range were determined with low confidence intervals (Table 2). Results show that the most potent compound is **124**, with an affinity for SerB2 almost twice as high as that of compound **91** and almost four times as high as that of compound **95**. Derivative **88** is the least efficient, which underlines once again the importance of the hydrophobicity and bulkiness of the substituents. Hill coefficients (n) were also calculated for the four systems and the values around 2 that were obtained suggest positive cooperativity in the binding of the inhibitor. That is, binding of a first molecule to SerB2 would facilitate the interaction with a second molecule by changing the affinity of the free binding site or creating it.

### 2.4. Induced Fit Docking and Binding Free Energy Calculation

**Figure 4:**
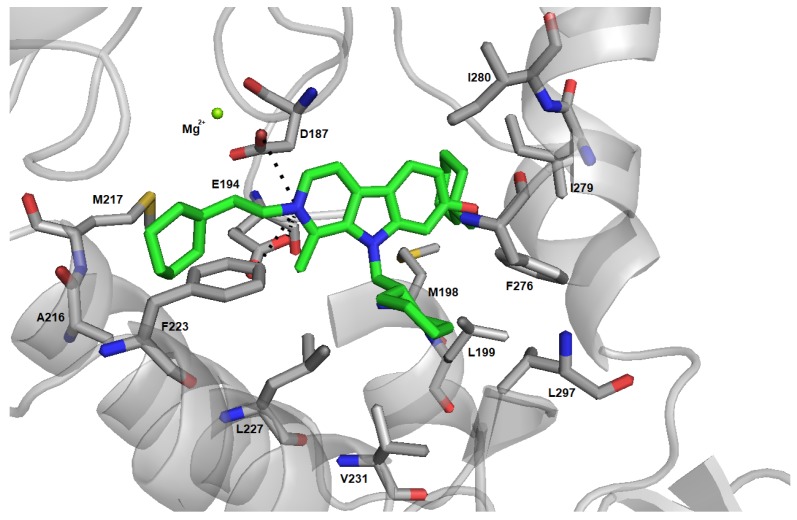
Structure of docked inhibitor **124** (green) within the active site of SerB2.

Considering the competitive nature of the inhibition kinetics mechanisms studied above, it is likely that inhibitors interact directly with the active site of the enzyme, disrupting substrate binding. Like other phosphatases, SerB2 possesses an open and a closed conformation in order to let substrates and products of the reaction in and out [55]. This kind of structure possesses highly dynamic properties already shown in similar enzymes [56,57,58]. The SerB2 model generated by homology modeling based on the crystal structure of *Mycobacterium avium* SerB (Protein Data Bank (PDB) entry 3P96) is in the closed conformation. Rigid docking of ligands as big as harmine derivatives was thus challenging. The Induced Fit Docking protocol was therefore chosen to take the mobility of the closing loop into account. All four inhibitors were successfully docked within the active site of SerB2 (Figure S1) with Glide XP scores ranging from −7.9 to −13.3 kcal/mol. The docked structure of the best inhibitor, compound **124**, is shown in Figure 4.

Analysis of those structures shows that all three substituents on the β-carboline core fill different pockets within the active site. Those pockets mainly contain hydrophobic residues like leucines, valine and isoleucines, explaining the selectivity for lipophilic substituents. Also, the substitution on position 2 is essential for activity as discussed in Section 2.1 since it introduces a positive charge on the N${}^{2}$ atom. The latter seems to form a salt bridge with the catalytic aspartate and a glutamate residue. There are also two phenylalanine residues within the active site that can form π-stacking and T-shape interactions with the β-carboline core or N-substituent of other derivatives (Figure S2).

Binding affinities of the four ligands were assessed using MM/GBSA calculations (Table 2). The ΔG${}_{bind}$ values show that **124** and **91** are the best inhibitors of SerB2 (with a slight preference for **124**). Compound **95** has less affinity for the enzyme since its value is 10 kcal/mol higher. Finally, compound **88** is predicted to be the less potent inhibitor of SerB2. Those calculations are in agreement with the experimental kinetics results.

### 2.5. MIC${}_{99}$ Determination Assays and Time-Dependent Bactericidal/bacteriostatic Activity

**Figure 5:**
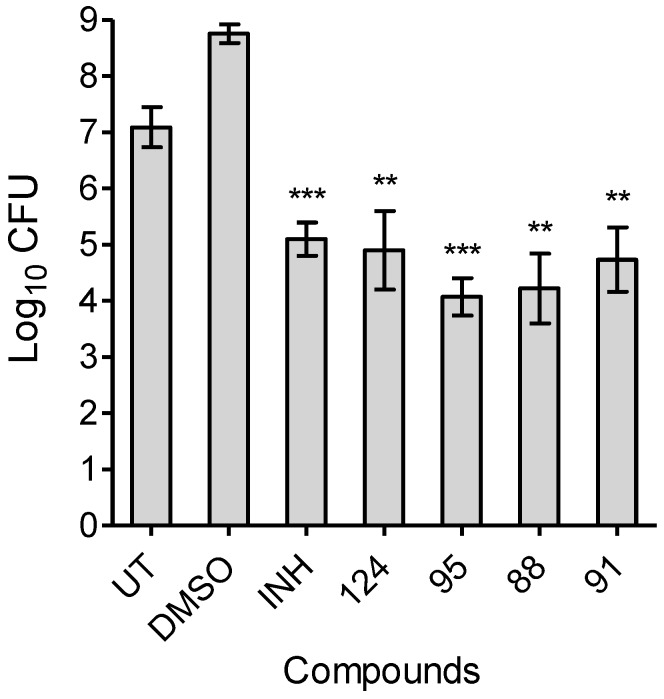
Time-kill kinetics of compounds **88**, **91**, **95**, **124** and isoniazid (INH) against *M. tuberculosis* H37Rv. Early logarithmic phase cultures of *M. tuberculosis* were exposed to 10 × minimal inhibitory concentrations (MIC${}_{99}$) of the compounds and bacterial enumeration (expressed in colony-forming units (CFU)) was performed by plating 10-fold serial dilutions on MB7H11 plates at day 6 post-exposure. The data shown is mean ± S.E. of data obtained from three experiments performed in duplicates. Statistical significance with respect to DMSO treated samples is reported as ** (*p* ≤ 0.01) and *** (*p* ≤ 0.001). UT: untreated.

In order to tackle the issue of drug-resistance, there is urgent need to identify drugs that target novel metabolic pathways and also possess bactericidal killing activity [59]. Compounds **88**, **91**, **95** and **124** were first evaluated for their ability to inhibit mycobacterial growth in vitro by determining their minimal inhibitory concentration (MIC${}_{99}$) against *M. tuberculosis* H37Rv. Values in the range 0.8–6.3 μM were obtained (Table 2). The results correlate well with the inhibition constants, which means that bacterial growth inhibition may be related to SerB2 inhibition. Next, time-dependent in vitro killing experiments were performed by exposing early-log phase cultures to 10× MIC${}_{99}$ of compounds **88**, **91**, **95**, **124** and isoniazid (INH). The activity of the hits was compared to untreated mycobacterial cultures as well as to INH exposed cultures (Figure 5). We observed that the selected compounds inhibited the growth of *M. tuberculosis* in a bactericidal manner. After 6 days of treatment, the range of killing observed in the case of the various harmine treated samples was comparable to those seen in the case of INH treated samples. Compounds **88**, **91**, **95** and **124** also appeared to be equally effective as no statistically significant difference was observed in their respective range of killing. As expected, no killing was observed in DMSO treated samples. These observations suggest that targeting of SerB2 by harmine derivatives results in bactericidal killing in vitro.

## 3. Conclusions

In this work, a first screening of 122 compounds from our internal chemolibrary led to the identification of a series of harmine derivatives targeting SerB2, an essential metabolic enzyme and suspected virulence factor of *M. tuberculosis*. Comparison of the best harmine-derived hits revealed that they share a common 7,9- or 2,7,9-substituted 7-oxy-1-methyl-β-carboline scaffold. A brief structure activity relationship evaluation led to the conclusion that this kind of substitution is essential for the inhibition of SerB2, as highlighted by the inactivity of harmine and harmol. Primary hits have been confirmed at 10 μM concentration and three 2,7,9-trisubstituted compounds were retained for further study.

In those three selected molecules (**88**, **91**, **95**), substituents are bulky aliphatic or aromatic groups. In order to verify whether the R${}^{2}$ substituent had to be aromatic for the molecule to achieve a high inhibition of SerB2, an original compound (**124**) has been designed, synthesized and tested. Compound **124**, bearing a N${}^{2}$-ethylcyclohexyl group that replaces the N${}^{2}$-ethylbenzyl group of compound **91**, showed similar activity in our enzymatic assay with inhibition at 10 μM of 93.5 ± 1.8 and 94.5 ± 1.4% for **124** and **91**, respectively. Therefore, it could be concluded that the aromaticity of the R${}^{2}$ substituent does not influence the inhibition of SerB2 and that its direct environment upon binding of the inhibitor is probably not made of aromatic residues.

Inhibition kinetics experiments were performed to further study the effect of **124** and the selected compounds. The shapes of the double-reciprocal plots and slope replots showed that all four compounds inhibit SerB2 in a parabolic competitive fashion with apparent $K_{i}$ values in the micromolar range. These experiments revealed that the most effective inhibitor was the newly synthesized **124** and confirmed the importance of hydrophobic and flexible substituents over aromatics. Hill coefficients were also calculated for the four molecules and indicate positive binding cooperativity. Along with the parabolic behavior, this interesting finding suggests that the interaction of a first inhibitor molecule with SerB2 facilitates the binding of a second one.

The interactions between the enzyme and the most potent derivatives were investigated by induced fit docking experiments and binding free energy calculations. Analysis of the simulated complexes suggests that all three substituents on the β-carboline core fill different pockets within the active site. Those pockets are mostly made of hydrophobic residues like leucines, valine and isoleucines, explaining the selectivity for hydrophobic substituents. Also, the third substitution appears essential for activity since it introduces a positive charge on the nitrogen atom of the pyridinium moiety. The latter could form a salt bridge with the catalytic aspartate and a glutamate residue. The binding free energy values calculated from the docking poses turned out to be consistent with the experimental data. Crystallographic studies are now underway to confirm those results.

Finally, the selected β-carbolines are also active against *M. tuberculosis* strain H37Rv in vitro with MIC${}_{99}$ values ranging from 0.8 to 6.3 μM. The correlation with the $K_{i}$ values suggests that SerB2 could be the main target of the tested compounds. Time-dependent killing experiments performed at 10× MIC${}_{99}$ of compounds **88**, **91**, **95** and **124** showed that they inhibit bacterial growth in a bactericidal manner. Altogether, these promising results may provide a basis for the development of new antimycobacterial agents targeting SerB2.

## 4. Materials and Methods

### 4.1. Expression and Purification of SerB2

*Escherichia coli* strains BL21 (DE3, pLysS) and BL21 (DE3) were respectively transformed with pET28b-*serb2* or pAVA0421-*serb2*. Both plasmids carry *serb2* gene under control of the T7 RNA polymerase promoter and encode for a N-terminal hexahistidine tag. The pET28b-*serb2* plasmid contains a gene for kanamycin resistance and its expression product was used in the screening of inhibitors. The pAVA0421-*serb2* was used for the kinetic studies and encodes for ampicillin resistance along with a human rhinovirus 3C (HRV 3C) protease site allowing cleavage of the hexahistidine tag. The cells were grown at 37 °C in LB liquid medium supplemented with the appropriate antibiotics until an optical density at 600 nm of 0.6–0.8. Protein expression was induced at 20 °C by the addition of 0.2 mM isopropyl β-d-1-thiogalactopyranoside (IPTG). The cells were grown for 18 h and harvested by centrifugation, resuspended in lysis buffer (50 mM Tris-HCl pH 7.4, 200 mM NaCl, 1 mg.mL${}^{-1}$ lysozyme, supplemented with cOmplete EDTA-free protease-inhibitor cocktail from Roche) and disrupted by sonication over ice. After centrifugation and filtration, the lysate was loaded onto a HisTrap FF crude IMAC column (GE Healthcare) and washed with buffer A (50 mM Tris-HCl pH 8.0, 200 mM NaCl, 20 mM imidazole). The protein was eluted with a linear gradient of buffer B (50 mM Tris-HCl pH 8.0, 200 mM NaCl, 500 mM imidazole). For the pAVA0421-*serb2* construct, the hexahistidine tag was removed by incubation with HRV3C protease overnight and separated from the cleaved protein by a second IMAC purification. The fractions containing SerB2 were dialyzed overnight at 4 °C against a storage buffer (50 mM Tris-HCl pH 7.4, 100 mM NaCl), aliquoted and flash-freezed for storage at −80 °C. Purity was verified by 12% SDS-PAGE stained with Coomassie Blue and concentration was determined spectrophotometrically at 280 nm using 11,710 M${}^{-1}$cm${}^{-1}$ as the extinction coefficient.

### 4.2. Enzymatic Assay, Screening of Inhibitors and Inhibition Kinetics Studies

Enzyme activity was assayed by Pi determination using a malachite green-based phosphatase assay adapted from the protocol of Arora et al. [22]. SerB2 is incubated at 37 °C in a 180 μL volume containing 25 mM Tris-HCl pH 7.4, 5 mM MgCl_2_, 1 mM DTT, 5% DMSO and the selected inhibitor (or no inhibitor for 100% activity control). The reaction is initiated by adding 20 μL 2 mM *O*-phospho-L-serine. After incubation for 10 min at 37 °C, the reaction is stopped by mixing 150 μL of the reaction volume with 50 μL dye composed of 1.7% ammonium heptamolybdate and 0.22% malachite green in 2 M HCl [47,48]. The absorbance of the solution is measured at 660 nm and the activity (released Pi) is calculated from a calibration curve constructed using dilutions of a phosphate standard solution. Tests are run in triplicate and include blanks run without *O*-phospho-L-serine. Inhibition kinetics measurements are performed following the same procedure with varying *O*-phospho-L-serine concentrations and fixed inhibitor concentration. For the analysis of kinetic data, initial velocities are reported against substrate concentration as the amounts of released Pi after 10 min reaction. In the screening and inhibition assays, inhibition percentages (inh%) are calculated according to Equation (1) where $n_{Pi}$ and $n_{Pi_{100\%}}$ are the amounts of released Pi for the inhibited condition and the 100% activity control respectively.

(1) $$inh\%=100-\frac{100\times n_{Pi}}{n_{Pi_{100\%}}}.$$

### 4.3. Evaluation of Kinetic Parameters

Analysis of kinetic data and curve fitting by linear and non-linear regression was performed using GraphPad Prism 5 (GraphPad Software, La Jolla California USA). Values of apparent K${}_{m}$ and V${}_{max}$ parameters were determined by fitting the Michaelis-Menten equation to the experimental velocity curves (*v* vs. [S]). Values of $K_{i}$ and n parameters were determined by fitting the slope function (Equation (3)) of the Hill-type inhibition model of Cao et al. [52] (Equation (2)) to the slopes${}_{LB}$ vs. [I] replots.

(2) $$v=\frac{V_{max}\times \left[S\right]}{K_{m}\left(1+\frac{{\left[I\right]}^{n}}{K_{i}^{n}}\right)+\left[S\right]}$$

(3) $$slope_{LB}=\frac{K_{m}}{V_{max}}\left(1+\frac{{\left[I\right]}^{n}}{K_{i}^{n}}\right).$$

### 4.4. Evaluation of Hydrophobic Parameters

Hansch hydrophobic parameters π were calculated using the Molinspiration online Property Calculation Service (www.molinspiration.com).

### 4.5. Induced Fit Docking

The structure of SerB2 was build from the known structure of *Mycobacterium avium* phosphoserine phosphatase SerB (84% identity, PDB entry 3P96) using Maestro Homology Modelling tool. The model was further minimized with the Protein Preparation Wizard [60]. The protonation of acid and basic residues was adjusted using Epik at a fixed pH of 7.4 [61]. The global structure was then refined with the OPLS3e forcefield. Inhibitors were prepared using the LigPrep tool of Maestro with the OPLS3e forcefield [62]. The Induced Fit Docking protocol [63,64] from Schrödinger’s 2019 suite was employed to dock tri-substituted harmine derivatives within SerB2’s active site. The receptor grid center was fixed on the magnesium ion and the cubic grid was set to dock ligands with length equal to 25 Å. Side chains were trimmed based on their B-factors during the initial docking phase and Glide XP precision was used for the final redocking step [65]. The affinity of ligands was then calculated using the Prime MM-GBSA [66,67,68] application from Maestro. This physics-based method is usually used to rescore ligands using the force field energies calculated for bound and unbound ligand in an implicit solvent model. The difference of energy between the enzyme-ligand complex and the unbound state of enzyme and ligand gives the ΔG${}_{bind}$ in kcal/mol. The VSGB solvation model was used with the OPLS3e forcefield.

### 4.6. MIC${}_{99}$ Determination Assays

*M. tuberculosis* H37Rv were cultured at 37 °C with continuous agitation at 200 rpm in middlebrook 7H9 medium supplemented with 0.2% glycerol, 1x Albumin-Dextrose-Saline (ADS), 0.05% Tween-80. For MIC${}_{99}$ assays, bacterial cultures were seeded at an OD${}_{600nm}$ of 0.0002 in 7H9 medium in the presence of varying concentrations of drugs in 96-well round-bottom plates (Nunc, USA). The last column wells of each plate were used for negative control (medium plus bacterial cells only). For positive control, antitubercular drug, INH, was used in each assay. Plates were incubated at 37 °C for 14 days. MIC${}_{99}$ was determined as the lowest concentration of drug at which no visible growth is observed.

### 4.7. Time-Dependent Bactericidal/Bacteriostatic Activity of Harmine Derivatives

Compounds **124**, **95**, **88** and **91** were selected for kill kinetics experiments against *M. tuberculosis* H37Rv. For killing experiments, early-log phase cultures were exposed to these drugs and isoniazid (positive control) at 10× MIC${}_{99}$ concentration for 6 days. At designated time points, samples were collected, diluted 10 folds and plated on middlebrook 7H11 medium at 37 °C for 3–4 weeks. To obtain time-kill curves, log10 CFU values were plotted against time (in days) using GraphPad Prism 5. Statistical analyses were performed using GraphPad Prism 5. Data were checked for homoscedasticity using a F-test before analysis. Data from each treated sample was compared with DMSO control data by performing an unpaired Student T-test (two-tailed). P values lower than 0.05 were considered statistically significant.

### 4.8. Synthesis

#### 4.8.1. General

All commercial starting materials were used without further purification. Technical solvents were distilled before use. Anhydrous THF was obtained by distillation under argon with sodium/benzophenone. Thin-layer chromatography (TLC) was performed on silica gel 60 F254 TLC plates (Merck) and revelated at 254 nm or by staining with p-anisaldehyde. Melting points were measured in open capillaries using a Büchi melting point B-450 apparatus. Mass spectra (MS) were recorded using electron spray ionization in positive mode on a Bruker maXis Impact spectrometer. Analytes were dissolved in dichloromethane (DCM) and injected at 3 μL/min. High performance liquid chromatography (HPLC) analyses were performed on an Agilent 1100 series HPLC using UV detection at 254 nm. The method used consisted of the injection of 10 μL of a 100 μg/mL solution onto an Agilent Zorbax Eclipse XDB C8 4.6 mm × 150 mm, 5 μm separation using a 30:70 solution of 0.01 M sodium butane sulfonate in water/ 0.01 M sodium butane sulfonate in methanol as the eluent (flow: 1.0 mL/min). ${}^{1}$H and ${}^{13}$C NMR spectra were recorded at 400 and 100 MHz respectively on a Jeol JNM ECX 400 spectrometer. Chemical shifts were reported in parts per million (ppm, δ) relative to the residual peak of the solvent (δ${}^{1}$H CDCl_3_ 7.26 ppm, δ${}^{13}$C CDCl_3_ 77.2 ppm, δ${}^{1}$H DMSO-d${}_{6}$ 2.50 ppm, δ ${}^{13}$C DMSO-d${}_{6}$ 39.5 ppm). Coupling constants are given in Hz. Proton coupling patterns are described as singlet (s), doublet (d), triplet (t), quartet (q), multiplet (m).

#### 4.8.2. Synthesis of 1-methyl-9H-pyrido[3,4-b]indol-7-ol hydrochloride dihydrate (Harmol)

Harmine (2.00 g, 9.42 mmol, 1 equiv) was dissolved in glacial acetic acid (40 mL) followed by addition of 47% aqueous hydrobromic acid (40 mL). The solution was refluxed for 72 h. After completion of reaction was confirmed by TLC, the mixture was diluted in water and concentrated under reduced pressure. Dilution and evaporation were repeated twice to afford a beige solid. Yield: 99%, R${}_{f}$: 0.37 (DCM/ethanol(EtOH): 85/15), mp 248 °C, MS [M+H]${}^{+}$ : 199.1, ${}^{1}$H NMR (400 MHz, DMSO-d${}_{6}$) δ (ppm) : 2.96 (s, 3H, CH_3_), 6.90 (dd, 1H, ${}^{3}$J = 8.70, ${}^{4}$J = 2.10, H-6), 7.04 (d, 1H, J = 2.10, H-8), 8.25 (d, 1H, J = 8.50, H-5), 8.35 (d, 1H, J = 6,18 H-3), 8.39 (d, 1H, J = 6,18, H-4), 10.46 (s, 1H, O-H), 12.52 (s, 1H, N-H), ${}^{13}$C NMR (100 MHz, DMSO-d${}_{6}$) δ (ppm) : 15.83, 96.49, 112.79, 112.88, 113.64, 124.78, 128.67, 132.43, 133.55, 136.53, 145.72, 161.35.

#### 4.8.3. 7-(cyclohexylmethoxy)-9(cyclohexylmethyl)-1-methyl-β-carboline (**123**)

Compound **123** was synthesized according to the general procedure described in Reference [42] from harmol hydrobromide dihydrate (1.50 g, 4.76 mmol, 1 equiv) in the presence of potassium hydroxide (1.34 g, 23.9 mmol, 5 equiv) and bromomethylcyclohexane (1.33 mL, 9.53 mmol, 2 equiv) in DMF (30 mL). A white solid was obtained after purification. Yield: 50%, R${}_{f}$: 0.75 (DCM/EtOH: 85/15), ${}^{1}$H NMR (400 MHz, CDCl_3_) δ (ppm) : 1.10-1.96 (m, 22H, cyclohexyles); 3.04 (s, 3H, CH_3_); 3.88 (d, 2H, J = 6.18, O-CH_2_); 4.28 (d, 2H, J = 7,10, N-CH_2_); 6.85 (d, 1H, J = 1,83, H-8); 6.87 (dd, 1H, ${}^{4}$J = 2.06, ${}^{3}$J = 8.47, H-6); 7.76 (d, 1H, J = 5.27, H-4); 7.95 (d, 1H, J = 8.47, H-5); 8.27 (d, 1H, J = 5.50, H-3), ${}^{13}$C NMR (100 MHz, CDCl_3_) δ (ppm) : 23.71; 26.00; 26.39; 26.66; 30.15; 31.19; 37.96; 40.36; 50.93; 74.09; 95.00; 109.20; 112.20; 114.88; 122.25; 129.63; 135.67; 138.11; 140.68; 143.96; 160.49, mp 148 °C, MS [M+H]${}^{+}$: 391.3.

#### 4.8.4. 7-(cyclohexylmethoxy)-9-(cyclohexylmethyl)-1-methyl-2-(2-cyclohexylethyl)-β-carboline -2-ium bromide (**124**)

Compound **124** was synthesized according to the general procedure described in Reference [42] from compound **123** (200 mg, 0.51 mmol, 1 equiv) and 1-bromo-2-cyclohexylethane (0.8 mL, 5.11 mmol, 10 equiv) in THF (7 mL). The purified product was a beige solid. Yield: 31%, R${}_{f}$: 0.23 (DCM/EtOH: 90/10), ${}^{1}$H NMR (400 MHz, CDCl_3_) δ (ppm) : 0.99-1.93 (m, 35H, N${}^{+}$-CH_2_-CH_2_-Cy, 2 cyclohexyles), 3.27 (s, 3H, CH_3_), 3.88 (d, 2H, J = 5.95, N-CH_2_), 4.37 (d, 2H, J = 7.10, O-CH_2_), 4.86 (t, 2H, J = 8.24, N${}^{+}$-CH_2_), 6.84 (d, 1H, J = 1.60, H-8), 6.95 (dd, 1H, ${}^{4}$J = 1.83, ${}^{3}$J = 8.93, H-6), 8.04 (d, 1H, J = 8.70, H-5), 8.23 (d, 1H, J = 6.64, H-4), 8.79 (d, 1H, J = 6,64, H-3), ${}^{13}$C NMR (100 MHz, CDCl_3_) δ (ppm): 16.79; 25.76; 25.88; 26.06; 26.30; 26.50; 29.77; 29.97; 31.06; 33.20; 35.44; 37.77; 39.06; 40.57; 51.96; 56.68; 74.30; 94.65; 112.87; 113.34; 114.77; 124.26; 133.93; 135.29; 135.29; 135.74; 136.97; 148.31; 163.66, mp 237 °C, MS [M+H]${}^{+}$ : 501.4, Purity: 100% by HPLC.

## Figures and Tables

**Table 1:**
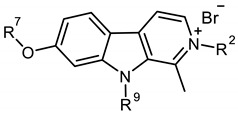
Structures of the common 2,7,9-substituted 7-oxy-1-methyl-β-carboline core and inhibition percentages at 10 μM (± S.E.) of SerB2’s phosphatase activity for the 7,9-disubstituted and ten 2,7,9-trisubstituted harmine derivatives that were primary hits in the screening. The Hansch hydrophobic parameters π of each substituent were calculated using Molinspiration and are given in the third and fifth column for $R^{7/9}$ and $R^{2}$ respectively. The compounds have been classified into three sets A, B and C according to the nature of their $R^{7}$ and $R^{9}$ substituents.

Set	Compound	R${}^{7}$/R${}^{9}$	R${}^{2}$	π(R${}^{2}$)	Inhibition at 10 μM (%)
A	39	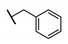 *π*(R^7^)/*π*(R^9^) 2.13/1.67	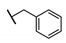	−0.02	0 ± 10.2
	86		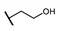	−2.25	0 ± 5.6
	92		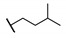	0.03	0 ± 7.6
	94		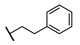	0.19	15.6 ± 4.3
	95		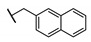	1.17	82.6 ± 9.9
B	87	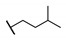 *π*(R^7^)/*π*(R^9^) 2.19/1.72	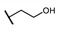	−2.25	0 ± 12.4
	88		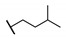	0.03	70.4 ± 9.1
	93		-	2.80	0 ± 17.4
C	89	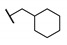 *π*(R^7^)/*π*(R^9^) 2.81/2.36	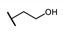	−2.25	25.1 ± 8.2
	90		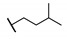	0.03	16.5 ± 13.3
	91		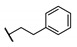	0.19	94.5 ± 1.4

**Table 2:** Inhibition kinetics parameters, estimated binding free energies ($\Delta G_{bind}$) and cellular activities of SerB2 inhibitors **88**, **91**, **95** and **124**. Inhibition constants ($K_{i}$) and Hill coefficient (n) values are reported with 95% confidence intervals. Goodness of fit of the Hill-type model used (Equation (3)) are reported as R${}^{2}$ values in the fourth column. $\Delta G_{bind}$ were calculated following induced fit docking experiments by the MM/GBSA method. Minimum inhibitory concentrations (MIC${}_{99}$) were determined against *M. tuberculosis* strain H37Rv in vitro.

Compound	$K_{i}$ (μM)	n	R${}^{2}$	$\Delta G_{\mathrm{bind}}$ (kcal/mol)	MIC${}_{99}$(μM)
**88**	18.3 ± 2.3	1.45 ± 0.38	0.985	−78.18	6.3
**91**	3.27 ± 0.12	2.39 ± 0.45	0.996	−108.72	1.5
**95**	6.66 ± 0.39	1.42 ± 0.16	0.998	−99.53	3.6
**124**	1.75 ± 0.11	1.78 ± 0.53	0.993	−111.80	0.8

## Supplementary Materials

Supplementary materials are available online at https://www.mdpi.com/1420-3049/25/2/415/s1.

## Abbreviations

The following abbreviations are used in this manuscript:

CFU	Colony Forming Unit
DCM	Dichloromethane
DMF	*N,N*-Dimethylformamide
HRV 3C	Human rhinovirus 3C
IMAC	Immobilized metal affinity chromatography
INH	isoniazid
$K_{i}$	inhibition constant
MIC	minimal inhibitory concentration
Mtb	*Mycobacterium tuberculosis*
PDB	Protein Data Bank
Pi	inorganic phosphate
PS	*O*-phospho-L-serine
TB	Tuberculosis
THF	Tetrahydrofuran
