# Supplementary material for: Identification and Repurposing of Trisubstituted Harmine Derivatives as Novel Inhibitors of Mycobacterium tuberculosis Phosphoserine Phosphatase
Source: Molecules. 2020 Jan 19;25(2):415. doi: 10.3390/molecules25020415 (PMC7024313; doi:10.3390/molecules25020415)
Supplement: Supplementary file 1 [file molecules-25-00415-s001.pdf]

Supplementary Materials: Identification and repurposing of trisubstituted harmine derivatives as novel inhibitors of *Mycobacterium tuberculosis* phosphoserine phosphatase

Elise Pierson [1,†,*], Marie Haufroid [1,†,*], Tannu Priya Gosain [2], Pankaj Chopra [2], Ramandeep Singh [2] and Johan Wouters [1]

A

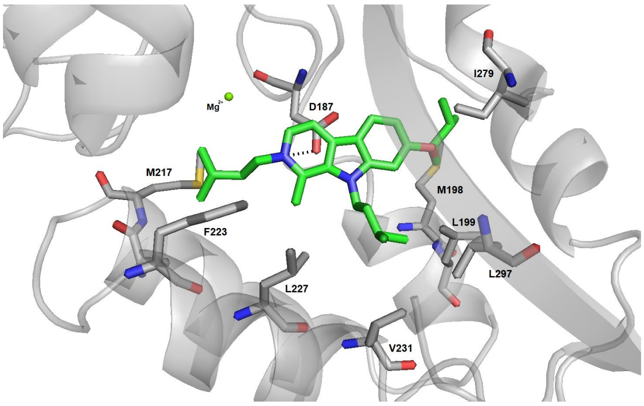

B

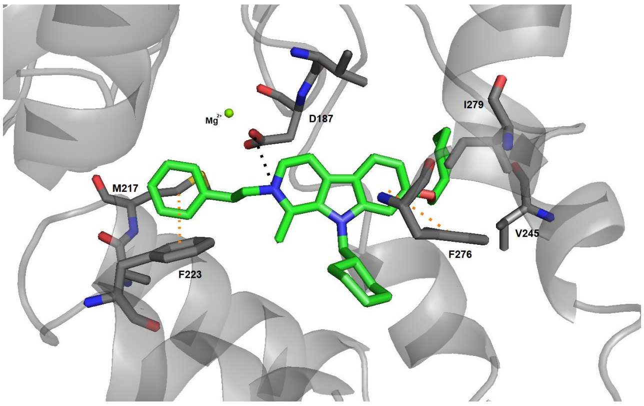

C

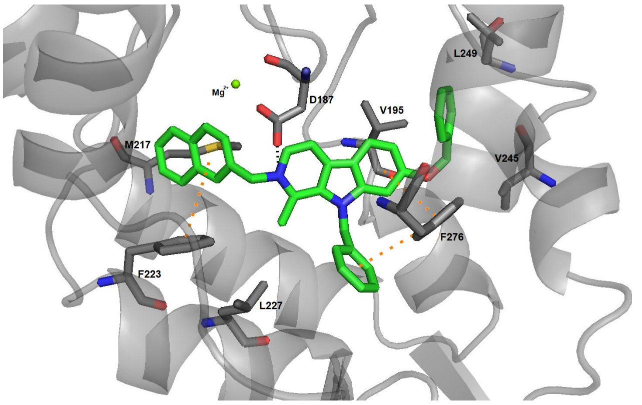

Figure S1. Structure of docked inhibitors (green) **88 (A)**, **91 (B)** and **95 (C)** within the active site of SerB2.

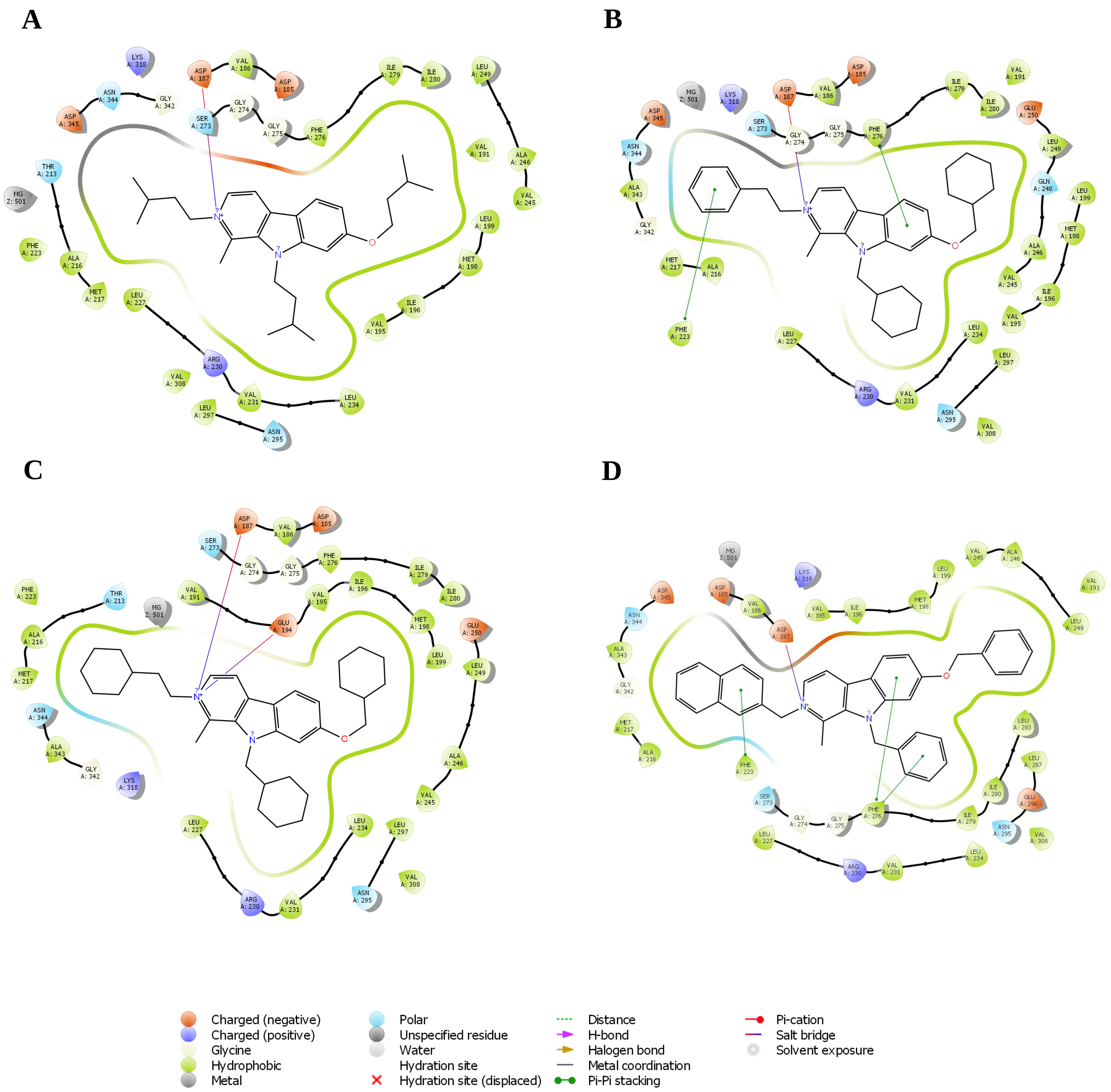

Figure S2. Two-dimension projection of docked compounds **88 (A)**, **91 (B)**, **124 (C)** and **95 (D)** within the active site of SerB2.
